# Investigating the Cytotoxicity of Folate-Conjugated Bismuth Oxide Nanoparticles on KB and A549 Cell Lines

**DOI:** 10.15171/apb.2018.071

**Published:** 2018-11-29

**Authors:** Fatemeh Akbarzadeh, Karim Khoshgard, Leila Hosseinzadeh, Elham Arkan, Davood Rezazadeh

**Affiliations:** ^1^Students Research Committee, School of Medicine, Kermanshah University of Medical Sciences, Kermanshah, Iran.; ^2^Department of Medical Physics, School of Medicine, Kermanshah University of Medical Sciences, Kermanshah, Iran.; ^3^Pharmaceutical Sciences Research Center, School of Pharmacy, Kermanshah University of Medical Sciences, Kermanshah, Iran.; ^4^Nano Drug Delivery Research Center, Kermanshah University of Medical Sciences, Kermanshah, Iran.; ^5^Medical Biology Research Center, Kermanshah University of Medical Sciences, Kermanshah, Iran.

**Keywords:** Cytotoxicity, Bismuth oxide, Nanoparticles, Folic acid, KB cells, A549 cells

## Abstract

***Purpose:*** Lately, bismuth-based nanomaterials have been widely utilized in medical researches such as imaging, drug delivery and radio-sensitization. Despite their advantages, bismuth-based compounds have shown toxic effects in humans. There are few studies on cytotoxicity effects of bismuth oxide (Bi2O3) nanoparticles (NPs) in-vitro. In this study, we aimed to investigate cytotoxicity of bare and also folate and 5-aminolevulinic acid (5-ALA)-conjugated Bi2O3 NPs on nasopharyngeal carcinoma (KB) and lung cancer (A549) cell lines.

***Methods:*** B_i2_O_3_ NPs were synthesized and conjugated with folate and 5-ALA. KB and A549 cells were cultured and incubated with 10, 20, 50 and 100 μg/ml concentrations of bare and folate-5-ALA-conjugated NPs. The survival rates were obtained after 2 and 24 hours incubation of the cells with NPs using MTT assay. Also, apoptosis and ROS generation induced by the NPs in the treated cells were obtained using Caspases-3 activity assay and flow cytometry analysis, respectively.

***Results:*** B_i2_O_3_ NPs were successfully synthesized with average size of 19.2 ± 6.5 nm, then conjugated with 5-ALA and folate. Either naked or folate-conjugated NPs were easily taken up by the cells in a concentration-dependent manner and showed cytotoxic effects. The significant cell death was noted at the concentrations more than 50 μg/ml for both compounds.

***Conclusion:*** Results indicated low cytotoxicity of the prepared NPs at lower incubation periods, which is very important for their further applications. However, 24 hours incubation of the cells with both forms of NPs caused more cell killing and the cytotoxicity increased with increasing concentrations of the NPs.

## Introduction

Recent advances in nanotechnology have led to the development of nanomaterials with potential of application in medicine, electronics, biosensors and biomaterials.^1-4^ The nanomaterials have unique properties that are completely different from their bulk forms.^5^ Due to their small size, nanomaterials have shown unique physical, optical, electronic, and chemical properties.^6,7^ For example, high-atomic number nanoparticles (NPs) such as bismuth (Z=83) oxide (Bi_2_O_3_) NPs have been proven to be radioenhancer in cancer radiotherapy.^8-12^ Moreover, bismuth does not accumulate in the body and therefore is biocompatible* in-vivo.*^11,12^ Due to its excellent biocompatibility and low cost compared to other high-Z nanomaterials such as gold, bismuth has recently been used as a high-sensitive contrast agent in CT-scan examinations.^13,14^

NPs usually induce toxicity when they enter into biological systems in medical applications.^15^ Surface modification methods such as PEGylation or silica coating, allow NPs to be utilized safely in biomedical treatments;^16,17^ however, such procedures are complex and time consuming.^18^ Folate (folic acid) has been used as conjugation agent to enhance cytotoxicity of NPs for cancer treatment.^8^ Folate receptors (FRs) are single-chain glycoproteins which possess high specific affinity for folic acid (FA) and are overexpressed on cell membrane of various malignant tumors,^19^ such as human oral squamous carcinoma (KB cells)^20^ but not in adenocarcinomic human alveolar basal epithelial cells (A549 cell line).^21^ Most of human normal cells have a little expression of FR, therefore the over-expression of FRs on membrane of the tumor cells can be exploited as a specific targeting ligand.^22^ This targeting strategy can be utilized for increasing the diagnostic and therapeutic efficacy in different types of cancers.

5-Aminolevulinic Acid (5-ALA) is an intermediate in heme biosynthesis pathway of cells. When exogenous 5-ALA is introduced into the cells, the concentration of protoporphyrin-IX (PpIX) increases during heme biosynthesis pathway.^23^ This PpIX can act as a strong photosensitizer in photodynamic therapy (PDT). In recent years, 5-ALA in conjugation with NPs has been used in PDT as an effective method for treating different kinds of malignant and non-malignant diseases.^24-26^

A few studies have been conducted to assess the potential cytotoxicity of Bi_2_O_3_ NPs before their biomedical applications.^6,27,28^ In this work, bare Bi_2_O_3_ NPs and folic acid-5-aminolevulinic acid (5-ALA-FA) conjugated Bi_2_O_3_ NPs were synthesized and their cytotoxicities at different concentrations were evaluated on KB and A549 cell lines *in-vitro.*

## Materials and Methods

### Chemicals and cell lines

The main chemical materials used for the synthesis of the NPs were bismuth(III) nitrate (≥98%)), propylene glycol (CH_3_CH(OH)CH_2_OH), reagent-grade ethanol, toluene (99.8%), ethyl-3-(3-dimethylaminopropyl) carbodiimide (EDC, ≥97%), N-hydroxysuccinimide (NHS, ≥98%), folic acid (FA; C_19_H_19_N_7_O_6_), (3-aminopropyl)trimethoxysilane (APTMS, 97%), and MTT (3-[4,5-dimethylthiazol-2-yl]-2,5-diphenyltetrazolium bromide) all purchased from Sigma-Aldrich (Missouri; USA); and dichloromethane, dimethylsulfoxide (DMSO), methanol (CH_3_OH), purchased from Merck (Darmstadt, Germany). The materials used for the cell cultures including Dulbecco’s modified Eagle’s medium (DMEM), fetal bovine serum (FBS), trypan blue, and trypsin-EDTA 0.25% all purchased from GIBCO (Invitrogen, Germany). The human KB cell line was kindly gifted by Dr Ali Shakerizadeh (Iran University of Medical Sciences, Tehran, Iran) and A549 cell line were obtained from Medical Biology Research Center at Kermanshah University of Medical Sciences (Kermanshah, Iran).

### Preparation of FA and 5-ALA-conjugated Bi_2_O_3_ NPs

Bi_2_O_3_ NPs were synthesized using bismuth nitrate according to previously published work of Luan, X et al.^29^ In brief, bismuth (III) nitrate was dissolved in propylene glycol and stirred overnight. Then, the mixture was placed in an oven at 150 °C for 3 hours to dry. Then synthesized NPs were functionalized as described by Bogusz. K et al.^30^ The synthesized Bi_2_O_3_ NPs, and toluene, were then poured into a schlenk flask; in which the mixture was exposed to N_2_ gas in one side and APTMS drop-wise (1 ml in 15 minutes) on the other side. After that, the mixture was sonicated, as well as temperature of the mixture increased to 50 ℃. Eventually the solution was stirred overnight. Next day, the sample was centrifuged at 5,000 rpm for 5 minutes. The sediment was washed with ethanol, and then dried at 60 ℃ during 5 hours. Afterwards, conjugating the NPs with 5-ALA and FA was performed;^31^ in which, 5-ALA was mixed with phosphate buffered saline (PBS). Then EDC was separately dissolved in PBS and added to the previous solution; then it was stirred for 20 minutes. NHS also was dissolved in PBS separately and added to the previous solution and stirred for another 20 minutes. The carboxyl group of 5-ALA got activated by this method; therefore, the prepared solution was added to the modified NPs followed by 30 minutes stirring. It should be noted that the modified NPs had been firstly dispersed in PBS. On the other hand, for activating the carboxyl group of the FA, it was dissolved in PBS. EDC was separately dissolved in PBS as well and added to the solution and stirred for 20 minutes. NHS was also dissolved in PBS and added to the solution, followed by another 20 minutes stirring. Activated FA was then added to the solution containing 5-ALA-conjugated NPs. Dialysis tubing was used for removing the salt and other impurities in solution in order to purify the final product.

### Structural characterization of the synthesized Bi_2_O_3_ NPs 

The size and shape of the Bi_2_O_3_ NPs were analyzed using transmission electron microscopy (TEM, Philips model CM30) operating at 150 KV. Samples were prepared on the copper grids by drop-coating of the NPs which were suspended in distilled water before analysis.

### FTIR Spectroscopy

FTIR spectra of Bi_2_O_3_ NPs and FA-conjugated NPs deposited in KBr disks were recorded on a IR Prestige-21 spectrometer (Shimadzu, Kyoto, Japan). Scanning was performed at room temperature, over the range of 4000 to 400 cm^-1^.

### Cell culture

KB cells (epidermal nasopharyngeal carcinoma, an established human cell line) and A549 cells (lung cancer cell line) were routinely grown monolayer using DMEM supplemented with 10% FBS in 25 cm^2^ flasks (SPL Life Sciences Co, Korea). The cell growth was carried out in a humidified atmosphere containing 5% CO_2_ and 95% air at 37 °C. When the confluency of cells reached more than 70%, they were washed with PBS and incubated with the trypsin-EDTA solution for 3 min at 37 °C to detach them from the flask. The cells were then re-suspended in the culture medium for seeding. Trypan blue staining was performed to determine the cell density before seeding.

### Cytotoxicity of the nanoparticles

The FR-positive KB and FR-negative A549 cell lineswere cultured in 96-well culture plates. When the cells reached to the confluency of more than 70%, they were treated with concentrations of 0 (control), 10, 20, 50 and 100 μg/ml of the FA-conjugated Bi_2_O_3_ NPs and also bare Bi_2_O_3_ NPs for 2 h and 24 h. Determining the concentration of FA-conjugated NPs was performed based on the existing NP concentration in the suspension of the compound. Afterwards, the medium was replaced with fresh medium containing 5% FBS. MTT assay was performed 24 h after incubation, in which 100 μl MTT solution was added to each well to reveal the viable cells. After further incubation for 2 h at 37 °C, culture medium of the wells was removed, and 100 μl DMSO was added to dissolve the formazan grains. The absorbance values of the wells were measured with an ELISA microplate reader (ELX 800, BioTek) at 490 nm wavelength.

### Determination of caspase-3 activity 

Together with MTT, caspase-3 activity assay was carried out to measure apoptotic cellular death induced by the synthesized NPs 24 h after incubation. The cells were seeded in 12-well plates and incubated with the FA-conjugated Bi_2_O_3_ NPs and also bare Bi_2_O_3_ NPs (0 as control, 10, 20, 50 and 100 μg/ml) for 2 and 24 h; then the cells were washed twice with PBS and incubated with DMEM containing 5% FBS. After 24 h, the cells were trypsinized and then centrifuged at 1400 rpm for 7 minutes. Thereafter, supernatant was removed and 30 μl lysis buffer was added to each sample and incubated on ice for 30 minutes and then centrifuged at 14000 rpm for 12 minutes. Afterwards, 5 μl of the supernatant was added to 30 ml of 1% reaction buffer/dithiothreitol (DTT) and mixed with 5 μl of 1 mM caspase-3 colorimetric substrate. The mixture was then incubated at 37 °C for 2 h and para-nitro aniline (p-NA) light emission was finally measured at 405 nm using a microplate reader. Protein concentrations were also equalized for each condition based on the Bradford method using the bovine serum albumin as a standard.

### Intracellular ROS measurement

The intracellular ROS level was measured using the cell permeable 2′,7′-dichlorofluorescin diacetate (DCFH-DA) dye which is non-fluorescent itself. It is oxidized when subjected to intracellular ROS and forms 2′,7′-dichlorofluorescin (DCF) which is a strong fluorescent compound. Briefly, a number of 5 × 10^5^ cells were cultured in each well of 12-well plates for 24 h. After 2 and 24 h exposures to different concentrations (0, 10, 20, 50, and 100 μg/ml) of the Bi_2_O_3_ NPs and FA-conjugated Bi_2_O_3_ NPs, the cells were washed with PBS and the cell culture medium was replaced with DMEM containing 5% FBS. After 24 h, the cells were stained with DCFH-DA for 45 min at 37 °C. Then, they were rinsed twice with PBS, trypsinized, centrifuged at 1000 rpm for 10 minutes and eventually resuspended in PBS. The fluorescence intensity of the treated cells was immediately analyzed using a NovoCyte benchtop Flow Cytometer (San Diego, USA).

### Statistical Analysis

All experiments were performed in triplicate and repeated at least three times. All results are presented as the mean ± standard deviation (SD). The level of cells' viability among different control and treated cell groups was compared using one-way analysis of variance (ANOVA) with a confidence interval of 95%.

## Results and Discussion

### Characterization of the synthesized Bi_2_O_3_ NPs

**Figure 1:**
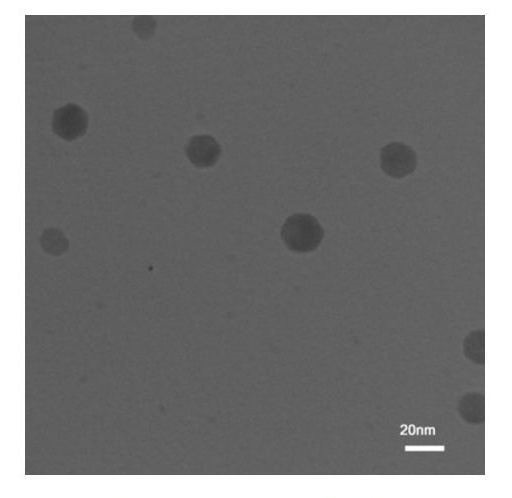
Transmission electron micrograph of the synthesized Bi_2_O_3_ nanoparticles.

Physical properties including morphology and size distribution of the synthesized Bi_2_O_3_ NPs were determined using TEM. Analysis of the NPs՚ size using the TEM images was performed on at least 400 NPs. TEM micrographs of the NPs showed the hexagonal or nearly hexagonal shape with an uniform size distribution (Figure 1). The mean ± standard deviation of the NPs' size was 19.2 ± 6.5 nm.

**Figure 2:**
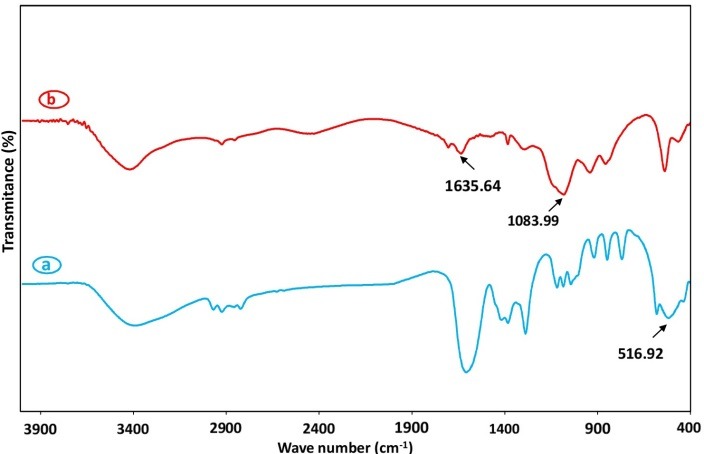
Fourier Transform Infrared Spectrum of (a) Bi_2_O_3_ nanoparticles and (b) Folate-conjugated Bi_2_O_3_ nanoparticles.

For verifying the conjugation process, Fourier-transform infrared spectroscopy (FTIR) spectra of the synthesized B_i2_O_3_ NPs and FA-conjugated NPs were done and the results are shown in Figure 2 a, b. The broad bandat 516.92 cm^-1^ in the FTIR of the NPs originates from the metal-oxygen (Bi-O) vibration and proves the NPs fabrication. Moreover, the peaks at 1635.64 cm^-1^ and 1083 cm^-1^ are characteristic of bending vibration mode of aromatic C=C of FA in FA-conjugated B_i2_O_3_ NPs.^32^

### Cytotoxicity of the FA-conjugated and bare Bi_2_O_3_ NPs

**Figure 3:**
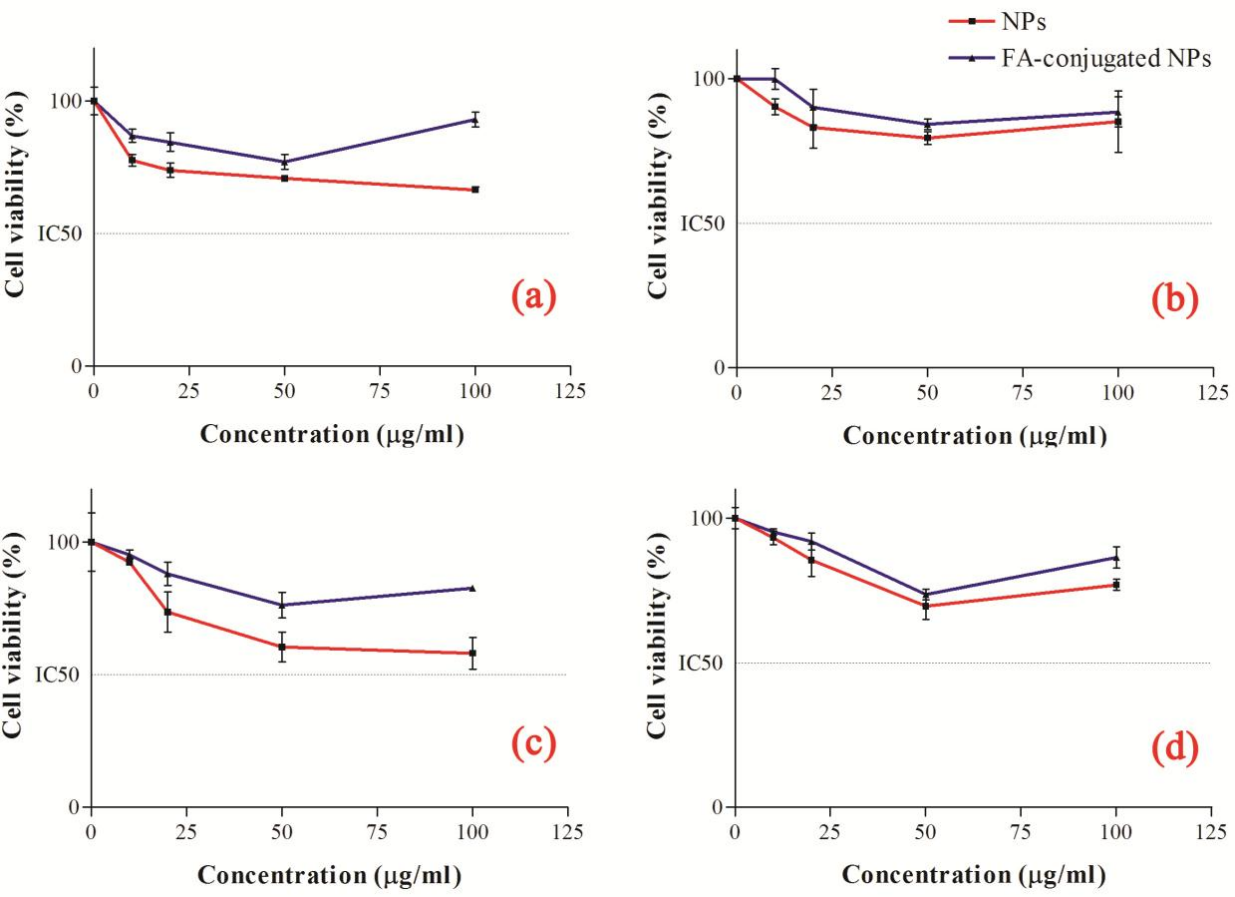
Cell viability of (a) KB and (b) A549 cells after 2 h and (c) KB and (d) A549 cells after 24 h incubation periods.

Few studies are performed to investigate toxicity of Bi_2_O_3_ NPs in mammalian cells.^6,27,28^ In the present work, the cytotoxicity of FA-conjugated Bi_2_O_3_ NPs and bare Bi_2_O_3_ NPs were evaluated using MTT, caspase-3 activity and flow cytometry assays on the KB and A549 cell lines; for which the examinations were performed after 2 and 24 h of exposure to 10, 20, 50 and 100 μg/ml concentrations of the NPs. Since the cytotoxicity of NPs depends on their concentrations, choosing appropriate concentrations in the toxicological studies is of great importance. Accordingly, the concentrations were chosen based on the former study in this issue.^27^Based on the MTT results, the dose effect curves were generated; and the concentration required to inhibit cell growth by 50% relative to the control (IC_50_) was obtained for both cell lines and with both type of the NPs. For 2 h incubation, viability of the cells did not change significantly after treatment with the NPs in the studied concentration range compared to control cells (Figure 3 a, b). However, 24 h incubation of the cells with both type of the NPs caused more cytotoxicity compared to 2 h incubation period (Figure 3 c, d) in a concentration-dependent manner. This finding is in agreement with previous work^27^ in which it has been shown that bare Bi_2_O_3_ NPs could dose-dependently increase the cell death by disturbing mitochondrial function in A549 cells.^27^ The low cytotoxicity of the Bi_2_O_3_ NPs at short incubation periods is very important specification which can be exploited for further applications. As shown in the graphs, cytotoxicity of the NPs is less than that to reach IC_50_ even in 24 h incubation periods.

KB cell line is well known as FR positive cells;^33^ due to overexpression of the folic acid receptors on the surface of these cells, FA-conjugated NPs are most probably internalized through receptor-mediated endocytosis.^22^ Therefore, this mechanism could increase internalization of the NPs into the cells through which may enhance the toxicity of FA-conjugated NPs on these cells.^34^ However, our findings indicate opposite results; as shown in Figure 3, cytotoxicities of the bare NPs in all groups are more than that of the FA-conjugated NPs while this effect cannot be seen in FR-negative A549 cells. As shown in Figure 3 b, d, cytotoxicity profile of the bare NPs and FA-conjugated NPs are quite similar and close together and there is no significant difference between these two treatment groups even in 24 h incubation period (P>0.05). Lower cytotoxicity induced by the FA-conjugated NPs compared to the bare NPs could be due to presence of 5-ALA in the FA-conjugated NPs compound. As described by Mohammadi *et al.,*^35^ 5-ALA increases the proliferation of the cells. As a matter of fact, this work is a part of our future study on photodynamic therapy of the KB and A549 cells, in which we will use the FA-5-ALA-conjugated Bi_2_O_3_ NPs as photosensitizing agents.

### Determination of caspase-3 activity 

In an study conducted by Abudayyak et al. the Bi_2_O_3_ NPs were found potent to increase the cellular death and induce apoptosis.^27^ The activation of caspase family proteins plays a key role in causing cell apoptosis. For this purpose, we investigated the activity of caspase-3 protein to determine potential of the FA-conjugated and also bare Bi_2_O_3_ NPs in apoptosis induction.

Colorimetric detection of para-nitro aniline (*p*NA) chromophore when it is cleaved from the substrate 7-Amino-4-(trifluoromethyl)coumarin-conjugated *p*NA in equal amount of cells lysis buffer is the basis of caspase-3 activity assay. In order to investigate the caspase-3 enzyme activity, cells were incubated with the FA-conjugated Bi_2_O_3_ NPs and also bare Bi_2_O_3_ NPs at concentrations of 0 (control), 10, 20, 50 and 100 μg/ml for 2 and 24 h; then pNA light emission was measured at 405 nm using a microplate reader.

**Figure 4:**
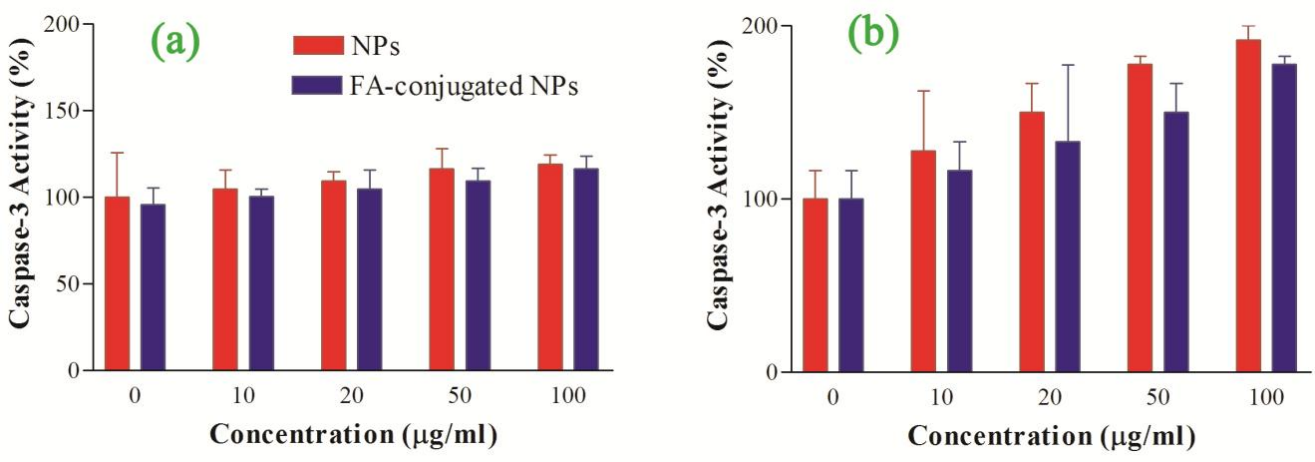
Caspase-3 activity in KB cell line after 2 h (a) and 24 h (b) incubation with 0, 10, 20, 50, 100 μg/ml concentrations of the bare and folic acid (FA)-conjugated NPs. The activity of caspase-3 enzyme was measured by colorimetric detection of para-nitro aniline and expressed as percentage compared to control (Mean ± Standard deviation).

**Figure 5:**
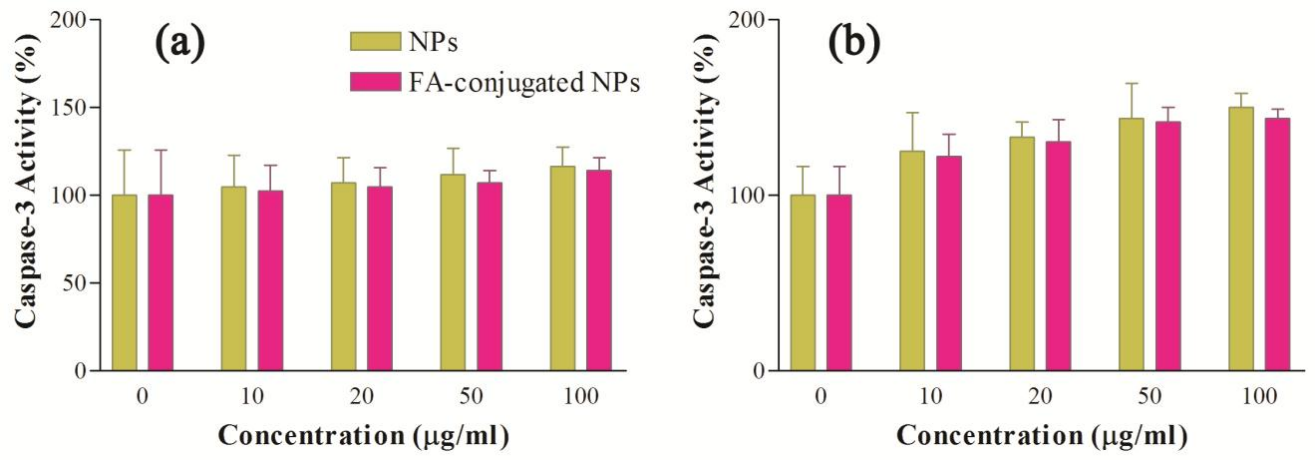
Caspase-3 activity in A549 cell line after 2 h (a) and 24 h (b) incubation with 0, 10, 20, 50, 100 μg/ml concentrations of the bare and folic acid (FA)-conjugated NPs. The activity of caspase-3 enzyme was measured by colorimetric detection of para-nitro aniline and expressed as percentage compared to control (Mean ± Standard deviation).

As shown in Figure 4 a, there is no significant difference in caspase activity of the KB cells between the control group and groups receiving either FA-conjugated or bare NPs after 2 h incubation period (P>0.05). Even at 100 μg/ml concentration of the FA-conjugated and bare NPs, the activity of caspase-3 enzyme increased up to about 19 ± 5.4% and 16 ± 7.5%, respectively. However, increasing the incubation period from 2 h to 24 h resulted nearly in 91 ± 8.3% and 77 ± 4.8%, enhancement in the caspase-3 activity at 100 μg/ml concentration of the FA-conjugated and bare NPs, respectively (Figure 4 b). In the case of A549 cells, 2 h incubating the cells with the FA-conjugated and bare NPs did not significantly change the activity of the caspase-3 enzyme (P>0.05) (Figure 5 a). However, 24 h incubating of these cells with the FA-conjugated and bare NPs at concentration of 100 μg/ml increased caspase-3 activity to 43.9 ± 5.3% and 50 ± 8.3%, compared to their control groups, respectively (P<0.05) (Figure 5 b).

The results indicate that both forms of the synthesized NPs can significantly increase caspase-3 activity compared to control group at large incubation periods in KB and A549 cells and thus can be utilized for cancer treatment approaches through induction of apoptosis.

### Effects of Bi_2_O_3_ NPs and FA-conjugated Bi_2_O_3_ NPs on ROS production

Bismuth oxide has a potential to either increase proliferation of cells through scavenging ROSs or increase cellular death rate through generating ROSs.^30^ Thus, to further support the cytotoxicity effects of the Bi_2_O_3_ NPs and FA-conjugated Bi_2_O_3_ NPs, their effects on the cells' viability were examined using flow cytometry method through detection of DCF fluorescence.

**Figure 6:**
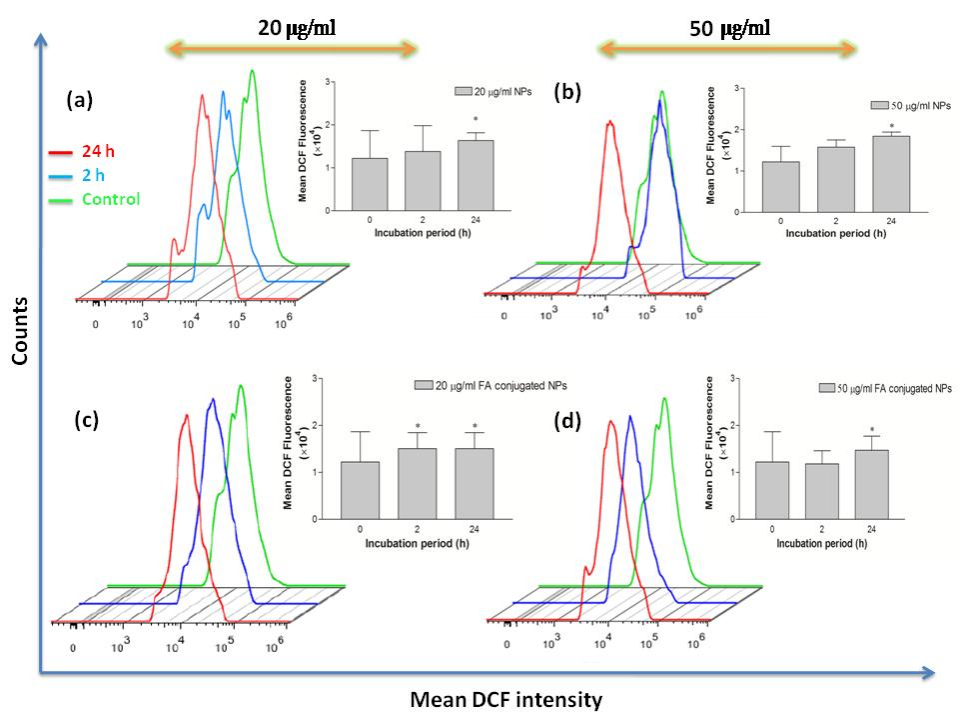
The enhancement of reactive oxygen species (ROS) generation in KB cells obtained with flow cytometry assay. The histograms demonstrate the intracellular mean fluorescence intensity when cells were treated with the bare and folic acid (FA)-conjugated NPs at different concentrations (0, 20, and 50 µg/ml) and different incubation periods (2 and 24 h). The data are expressed as mean ± standard deviation (n = 3), *p˂0.05 versus control group.

**Figure 7:**
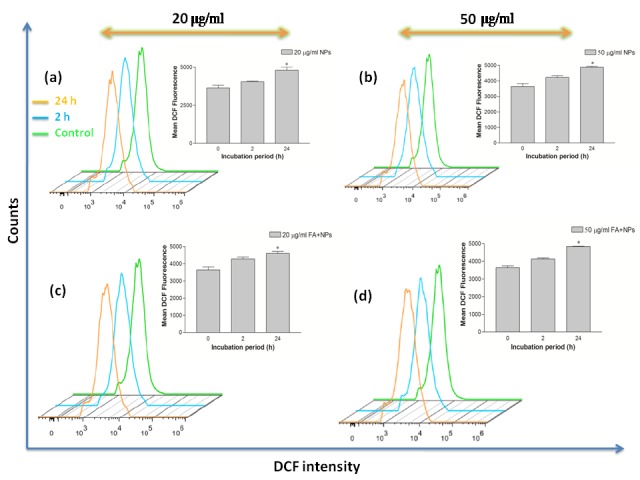
The enhancement of reactive oxygen species (ROS) generation in A549 cells obtained with flow cytometry assay. The histograms demonstrate the intracellular mean fluorescence intensity when cells were treated with the bare and folic acid (FA)-conjugated NPs at different concentrations (0, 20, and 50 µg/ml) and different incubation periods (2 and 24 h). The data are expressed as mean ± standard deviation (n = 3), *p˂0.05 versus control group.

Potential of the tested NPs in ROS generation are illustrated in Figures 6, 7. According to the results, a significant increase in DCF signal was found when the incubation time prolonged from 2 h to 24 h (*P<0.05) in both cell lines. This proves that Bi_2_O_3_ NPs can generate ROS which is able to damage the DNA and induce the cellular death.

**Figure 8:**
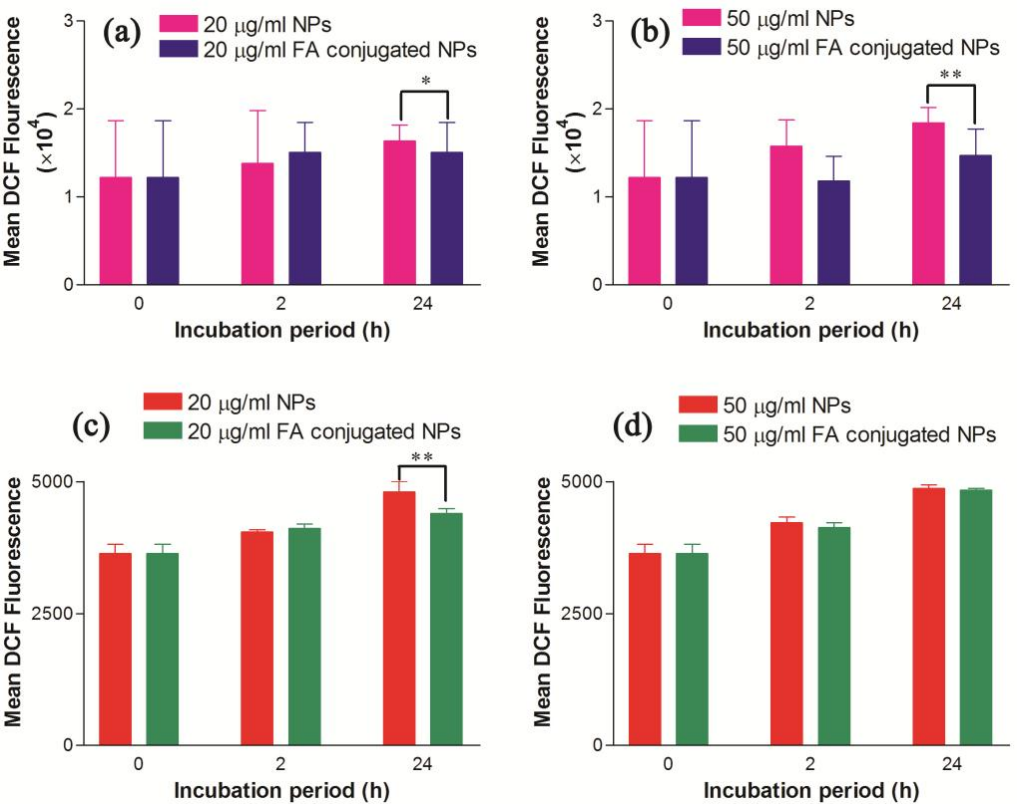
Reactive oxygen species (ROS) generation in KB cells (a, b) and A549 cells (c, d) caused by 2 and 24 h incubation of the cells with 20 and 50 μg/ml concentration of bare and folic acid (FA)-conjugated nanoparticles. The data are expressed as mean ± standard deviation (n = 3), **p˂0.01 and *p˂0.05 versus control group.

Both forms of the NPs showed different levels of ROS production in KB and A549 cells. In agreement with the MTT results, higher DCF signal is detected in the cells treated with Bi_2_O_3_ NPs (Figure 8). This strongly validates oxidative stress contributed to the cytotoxicity of these NPs. Regarding Figure 8, the flow cytometry assay of the FA-conjugated Bi_2_O_3_ NPs at the concentration of 50 μg/ml, revealed a decrease in ROS signal compared with uncoated Bi_2_O_3_ NPs which is more significant in KB cells than A549 cell line. Together with the cell proliferation induction effect of 5-ALA,^35^ this result may be explained by shielding effect of APTMS in FA-conjugated NPs in which the NPs are coated by APTMS for the purpose of conjugating 5-ALA and FA. According to previous studies, the cytotoxicity of the NPs could be lessened when they are coated by APTMS.^30^ However, this difference in ROS generation potential of bare NPs and FA-conjugated NPs was not significant in A549 cells at the concentration of 50 μg/ml (Figure 8 d).‏

## Conclusion

Bi_2_O_3_ NPs have been successfully synthesized and then conjugated to 5-ALA and FA in the present study. The cytotoxicities effects of both of the synthesized NPs were studied on KB and A549 cells using three assays including MTT, caspase-3 activity, and flow cytometry. The results showed a significant cytotoxicity in treated cells so that the FA-conjugated NPs had lower cytotoxicity than bare Bi_2_O_3_ NPs for either 2 or 24 hincubation periods in KB cell line. The results showed that conjugating the Bi_2_O_3_ NPs with FA and 5-ALA reduces their cytotoxicity in KB tumor cells; while similar cytotoxicity profiles were seen in FR-negative A549 cells using both forms of NPs. Based on the results, such NPs can be exploited for targeting the FR positive cancer cells such as human nasopharyngeal epidermal carcinoma. The utilized FA targeting ligand in our study is relatively effective targeting agent, but it may not be the best candidate to target other cancerous cells; therefore, further studies could be performed on such cancer cells with more specificity in targeting such as antigens and aptamers for diagnosis and treatment purposes.

## Acknowledgments

The authors gratefully acknowledge the research council of Kermanshah University of Medical Sciences (Grant Number 95678) for the financial support. This work was performed in partial fulfillment of the requirements for the Master of Science degree of Fatemeh Akbarzadeh, in the School of medicine, Kermanshah University of medical sciences, Kermanshah, Iran.

## Ethical Issues

Not applicable.

## Conflict of Interest

The authors declare that there are no conflicts of interest.
